# Hierarchical Human-Inspired Control Strategies for Prosthetic Hands

**DOI:** 10.3390/s22072521

**Published:** 2022-03-25

**Authors:** Cosimo Gentile, Francesca Cordella, Loredana Zollo

**Affiliations:** 1 Unit of Advanced Robotics and Human-Centred Technologies, Università Campus Bio-Medico di Roma, 00128 Rome, Italy; f.cordella@unicampus.it (F.C.); l.zollo@unicampus.it (L.Z.); 2 INAIL Prosthetic Center, Vigorso di Budrio, 40054 Bologna, Italy

**Keywords:** prostheses, prosthetic, hand, human-ispired, control, strategy, level

## Abstract

The abilities of the human hand have always fascinated people, and many studies have been devoted to describing and understanding a mechanism so perfect and important for human activities. Hand loss can significantly affect the level of autonomy and the capability of performing the activities of daily life. Although the technological improvements have led to the development of mechanically advanced commercial prostheses, the control strategies are rather simple (proportional or on/off control). The use of these commercial systems is unnatural and not intuitive, and therefore frequently abandoned by amputees. The components of an active prosthetic hand are the mechatronic device, the decoding system of human biological signals into gestures and the control law that translates all the inputs into desired movements. The real challenge is the development of a control law replacing human hand functions. This paper presents a literature review of the control strategies of prosthetics hands with a multiple-layer or hierarchical structure, and points out the main critical aspects of the current solutions, in terms of human’s functions replicated with the prosthetic device. The paper finally provides several suggestions for designing a control strategy able to mimic the functions of the human hand.

## 1. Introduction

Thousands of years ago, Aristotle described the hand as follows: «For the hands are instruments or organs, and the invariable plan of nature in distributing the organs is to give each to such animal as can make use of it [...] man does not owe his superior intelligence to his hands, but his hands to his superior intelligence. For the most intelligent of animals is the one who would put the most organs to use; and the hand is not to be looked on as one organ but as many; for it is, as it were, an instrument for further instruments» [1].

A child learns about the world through the hands even before using the other senses. Human beings develop new skills with their hands and use them for every daily action.

A study about the hand was carried out by Sir Charles Bell in 1834 [2], who analyzed the hand starting with a comparison with animal anatomy. In 1900, two famous anatomists, Frederic Wood Jones [3] and Russell Napier [4], studied the primitive nature of the human hand and the similarity with the other pentadactyl mammals’ upper limbs, remarking that functions as prehension or dexterity belong only to primates and humans [5]. The hand has always fascinated many people, from scientists to artists, and many studies have been conducted to describe and understand a mechanism so perfect and important for human activities.

The hand is one of the most important parts of the human body, used to learn and to interact with the environment. Therefore, hand loss represents irreparable damage for a person: life is upset, activities of daily living (ADLs) are compromised. Besides having suffered hand loss, the amputee will have to learn to perform everyday life actions with only one hand. To remedy this problem, since ancient Egypt, prostheses have been used both for cosmetic and functional purposes [6]. The first documented amputee who used a prosthetic limb is General Marcus Sergius, who lived in Ancient Rome [7]: «In his second campaign Sergius lost his right hand. [...] He had a right hand made of iron for him and, going into battle with this bound to his arm, he raised the siege of Cremona, saved Placentia and captured twelve enemy camps in Gaul—all of which exploits were confirmed by the speech he made as praetor when his colleagues tried to debar him as infirm from the sacrifices» [8].

The process of prostheses development began over 2000 years ago, but the first externally powered prosthesis was made only in 1919, using pneumatic and electric power sources [9]. The first myoelectric prosthesis was made in 1948 [10], a simple device with actuators powered using the amplified superficial electromyographic signals (sEMG). However, this idea had no future either in clinical and in commercial fields until 1969 when it was reinvented [11].

Nowadays, the use of EMG signals is the most common approach to actively control prosthetic hands [12]. After 1948, many research units developed myoelectric control in complete autonomy, reaching comparable results among them. A simple solution using EMG signals is the on/off control, i.e., when the signal exceeds a threshold, an output is sent to the prosthetic hand motors [13]. In [14], two electrodes were placed on agonist and antagonist muscle pairs, so a single motion (opening and closure) is associated with a single muscle. Another solution envisaged the use of EMG dynamic to proportionally modulate force or speed [15,16]. The number of degrees of freedom (DoFs) is small; a solution to overcome this limitation is offered by pattern recognition [17,18,19]. This technique extracts several features from different time segments of sEMG and uses them as input to the classifier to predict different grasps. The classifier output can be used to control a prosthetic device.

Many amputee subjects do not use myoelectric prostheses because their control is unnatural and not intuitive [20]. Unfortunately, the actual commercial prostheses are typically driven with proportional or on/off control [13], with a limited number of grasping configurations. Commonly, just the opening and closing of the prosthetic hand are possible, by using sEMG signals related to the flexion and extension of the wrist [21]. In the last 30 years, technologies and functioning of commercial prosthetics have not substantially changed [22], resulting in the abandonment of the artificial hand [23,24]. Marginal functional improvement in daily life is offered by the commercial prostheses [25], whereby the rejection reaches 59% (for amputations close to the wrist [26]) or 75% (for myoelectric prostheses [27]). Commercial prostheses are perceived as extraneous devices and not like the lost limb [28]. More realistic rates for rejection and non-usage have been estimated to be even higher due to the lack of contact between the clinic community and non-users [29]. An interesting result comes from the CYBATHLON 2016 [30], where competitors with prosthetic arms attempted both fast and precise manipulations performing a generic task choice based on ADL. The winner of the competition wore a simple body-powered arm.

A study carried out in [31] analyzed the real needs of the amputee subjects and provided insights into the development of prostheses more similar to the human hand. The fundamental demands emerging from this study are: to carry out ADLs, to have sensory feedback, to regulate force during grasp lightening the visual attention and the cognitive burden for the user, to avoid the slippage of the grasped object [32], to manipulate objects, and to handle small objects.

Leaving aside the recovery of sensory feedback in amputee subjects, the other issues can be addressed by improving the current control strategies.

The functioning of an active prosthetic hand is guaranteed by (i) a mechatronic device, with sensors and actuators, (ii) a system decoding human biological signals into gestures and (iii) a control law to translate all the inputs (from the hand and the user) into desired movements [33].

A control law replacing human hand functions and making a prosthesis acceptable and simple to use by the amputee [34] is the real challenge.

## 2. Aim of the Study

This paper intends to carry out an in-depth study of the literature on control strategies for prosthetic hands with multiple layers or a hierarchical structure to consolidate current knowledge in this field and highlight the lack of a control strategy allowing stability and usability during a simple grasping task, encouraging the prosthesis acceptability by the amputee. This work has the twofold purpose of (i) focusing research efforts toward the development of control strategies for hand prostheses replicating the performance of the human hand; (ii) providing foundations for future studies to in-depth explore the neurophysiological behavior of a limb related to the hierarchical management of the prehension aimed to replicate its functioning on a robotic device. The expected added value provided by this work is to update the current knowledge of control strategies with more recent papers, by critically evaluating and (possibly) comparing the available results and pointing out inconsistencies and neglected aspects. Indications for the development of future strategies for making hand prostheses appealing to individuals with hand loss are also provided.

The paper is organized as follows. Section 3 describes the methods used to select the reviewed articles. Section 4 introduces an overview of the control laws for prosthetic hands. Section 5 reports the hand functioning useful to understand the information to use in the development of prostheses control strategies. Section 6 describes the control strategies used in the analyzed papers. Section 7 underlines the principal limits of the current control strategies and suggests a methodology to develop new control strategies. Finally, conclusions are drawn in Section 8.

## 3. Methods

An extensive literature analysis was carried out on the following databases: PubMed, Google Scholar, IEEE Explore, and Scopus. The keywords (and their combinations) adopted for the research are the following: control strategy, upper limb, prosthesis, prosthesis control, grasping, pre-shaping, hierarchical control, multilevel control, and prehensile control. All publications in English appearing between 1960 and 2021 were considered. Moreover, from the selected papers, bibliographies were examined for extracting additional papers.

**Figure 1:**
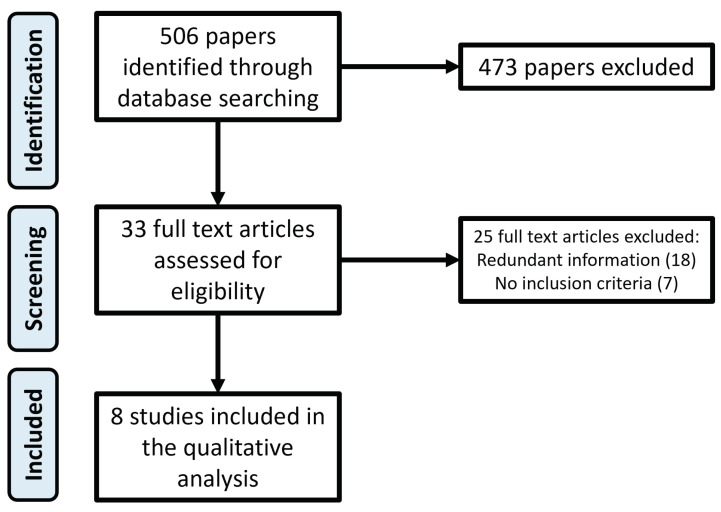
Flowchart of the search and inclusion process.

The inclusion criteria for selecting the publications relevant for the review purpose are as follows: control strategies for prosthetic hands, prehensile control of a prosthetic robotic hand, pre-shaping and grasping phases for controlling prosthesis, reach, and grasp. A flowchart of the search and inclusion process is shown in Figure 1. The result of applying the described method was 506 papers. They were evaluated by applying the addition criteria from the multilevel strategy and hierarchical strategy. After analyzing the title and abstract, all irelevant papers were discarded. From the initial 506 papers, 473 were excluded because they were considered not relevant and 33 papers have been carefully read. Twenty-five of these have been further excluded because they reported redundant information or did not meet the inclusion criteria.

The authors reviewed the remaining eight papers fulfilling the inclusion criteria. In particular, each analyzed paper must describe:

1. A control strategy for prosthetic hands that mimics the human hand behavior;
2. A control strategy with complete management of the different phases of the grasp;
3. A control strategy with multiple-layers o hierarchical structure;
4. Be a full-length publication in a peer-reviewed journal or conference proceedings.

## 4. The Beginning of Control Laws for Prosthetic Hands

Myoelectric prosthetic hands were initially inspired by robotic hands, focusing on the experience achieved with them, as reported in [31,35,36]. In [37,38], the first computer-operated mechanical hand and a robotic hand that can be considered the first dexterous multi-finger hand have been presented, respectively.

Once an object in a stable grasp moves due to some disturbance, a control system is necessary to allow the prosthetic hand to avoid the loss of the contact points between the hand and the grasped object. The successive step is applying the correct force on the object to be grasped and manipulated, guaranteeing grasp stability.

The development of both prosthetic hands and control strategies has been pursued in parallel, as shown in [38] where in addition to the hand, a control strategy was developed to mimic human reflexes and in [39] where a sequential controller made of relay circuits was realized to drive a three-fingered hand. In particular, these control methods emphasize reaction rather than stability.

Sensors play a significant role in control development since providing information about positions, forces and torques. The information from positions, forces, and torques allow realizing control strategies to regulate forces and to avoid object slippages during grasping [40].

The first prosthetic hands tried to imitate the behavior of human hands but due to the limitation of technology and interfacing systems, they looked more like robotic grippers. In the last 50 years, many control strategies were developed, exploring several scenarios to overcome these limitations.

Until the 1990s, hardware and control strategies were developed without considering the human hand as an inspiration, but around the 2000s there was a countertrend. Indeed, the properties of the human hand, such as the opposition of the thumb [41] or the postural synergies for the dimensionality reduction of hand DoFs [42], were introduced to develop bio-inspired controllers and hand structures [43].

Empirical approaches based on the imitation of human grasping strategies have been proposed [44] to reduce the computational burden of grasp control. In particular, a study on whether humans use a combination of basic grasp configurations has been performed to facilitate the replication of human-like behavior on robotic devices [45].

The development of a device replicating human behavior requires knowledge of that behavior. In particular, the computational burden of control approaches needs to consider the physiological reaction time of the human hands, useful to perform a simple task (i.e., between 50 and 100 ms) [46,47,48]. Therefore, the controller cycle velocity should also consider this aspect, avoiding too high values which could not make natural the use of prostheses. In particular, prostheses with control strategies with too rapid responses may not be managed by the subject who has slower reaction times, affecting the naturalness of the action.

The human hand behavior should be the basis for the development of a control strategy to be applied on prosthetic hands. Therefore, the first step to making an active prosthesis inspired by the human hand is understanding the motor control of the hand by the brain [49]. The evolution of neurosciences allowed the intensification of the study of hand functioning.

## 5. Human Hand Functioning

A study performed by analyzing the brain could describe in detail the real functioning of the human hand. First studies were performed on the primate brain to find some similarities with that of humans.

Thanks to innovative techniques [50,51,52,53], non-invasive studies were carried out on humans allowing finding similarities and differences in brain activity between macaques and humans [54,55,56,57,58] bringing out the grasping mechanism is based on the properties of the object to be grasped [55], including weight, surface, etc. [59,60,61,62,63,64]. Furthermore, the choice of the grasping configuration is affected by the task to be performed with the grasped object [65,66,67,68].

**Figure 2:**
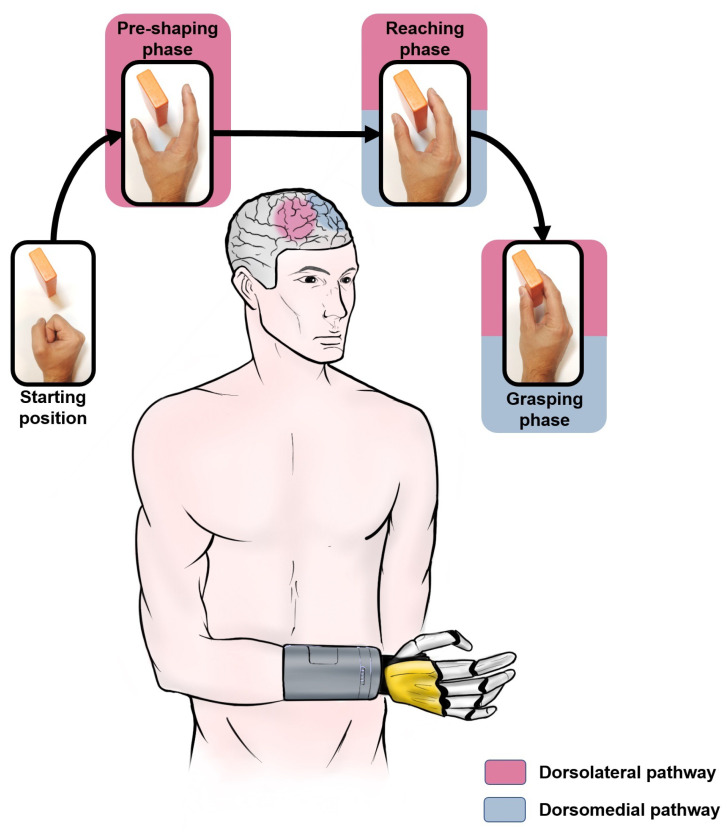
Activation of brain areas during prehension.

Information, as electrical impulses, travel from one region to another of the nervous system through a series of connected nerves formed by axons making synapses among neurons [69]. The flow of information related to the various phases of prehension is allowed by two pathways: the dorsolateral, to code the grasping, and the dorsomedial, to code the reaching (Figure 2).

The first one connects the anterior part of the intraparietal sulcus (AIP) [70] until the inferior parietal lobule (IPL) and the F5 area until the ventral premotor cortex (PMv) [71,72]. This pathway is involved in the motor commands for the hand pre-shaping, by transforming the grasped object proprieties (e.g., texture, size, etc.), derived by the visually guided grasping [73], in the corresponding commands to open the hand.

The second one connects two regions within the posterior parietal cortex (PPC), area V6A [74] and medial intraparietal area (MIP) [75], with the dorsal premotor cortex (PMd [76]). This pathway integrates somatosensory and visual information [55] for planning and controlling arm position during the transport phase.

However, a specific pathway subdivision is not possible because the functioning of each phase happens with an overlapping of the different areas [77,78,79,80]. The core region in the dorsomedial pathway codes information for grasping and reaching. In the same way, some regions between the two pathways code reaching information. The areas forming the pathways are highly distributed and the overlapping moves to the desired hand movement with a gradient [81]. Nevertheless, presently, many studies are still focused on this mechanism to find out how prehension works in humans.

Despite the lack of a complete explanation of the neurophysiological behavior for the prehension, the development of some control strategies would be possible by inspiring the information derived from these studies [82].

### 5.1. Tactile Sensory Mechanisms

During object manipulation, the human brain uses tactile information related to contact forces, the shape of the surfaces, and friction between the object surface and the fingertips.

The glabrous skin of the hand is equipped with about 17,000 sensory units sensitive to mechanical skin deformation and represents the enormous capability for spatial and temporal discrimination in this skin area [83]. These sensory units are of four types with distinctly different response properties: two fast adapting (FA-I, FA-II) and two slowly adapting (SA-I, SA-II) [84,85,86].

FA-I and SA-I afferents terminate superficially in the skin, with a particularly high density in the fingertips. FA-Is, connected to Meissner endings, exhibit sensitivity to dynamic skin deformations of relatively high frequency [87,88]. A single FA-I unit elicits a sensation of touch [83]. SA-Is, connected to Merkel cells, are most easily excited by lower-frequency skin deformations [87,88].

FA-II and SA-II afferents innervate the hand with a lower and roughly uniform density and terminate deeper in dermal and subdermal fibrous tissues [83,84,85,86]. The sensitivity of FA-II units, presumably connected to Pacinian corpuscles, is extremely high for skin deformation, particularly for rapidly moving stimuli [83,84,85]. The SA-II units, presumably connected to the spindle-shaped Ruffini ending, respond to direct skin indentations and to the skin stretching which normally occurs during the joints movements [83,84,85]. Moreover, during the manipulation of an object with the hand, SA-II units respond to the tangential forces in the skin and can provide information for controlling the grip force to avoid slipping, eliciting a reflex response in the muscle [86].

### 5.2. Grasp Stability

When moving and manipulating an object, the fingers involved in grasping apply tangential forces to the object surface while they apply normal forces on it to ensure grasp stability [89,90,91,92,93,94]. The grip force control is based on the prediction of the dynamic properties of the objects influencing the mapping between motor commands of the arm and resultant tangential forces and torques [95,96,97,98]. Dexterous manipulation involves balancing grip and load forces with object surface properties, a capability lost with an amputation. Indeed, healthy people regulate grip and load forces according to different frictional conditions, using high grip forces with more slippery surfaces [89,90,91,92,93,99]. Similarly, people adjust grip and load forces to the shape of the object to ensure grasp stability [90,100,101]. The result of these adaptations avoids an excessive grip force. The responses of the tactile afferents at the initial contact provide information about surface properties. A mismatch between predicted and actual sensory information can trigger corrective actions, leading to changes in grip-to-load force ratios after ~100 ms from the contact and giving place to an updating of the representation of the surface properties used in future interactions with the object [68,102]. Visual cues about the object shape can provide the information required to make predictions [100,101], but shape information provided by tactile signals after contact can override predictions based on visual cues.

### 5.3. Link between Brain Organization and Prosthesis Control Levels

The previous paragraphs describe the functioning of the hand during grasping. Different human brain areas manage each grasping phase:

- object recognition;
- object properties transformed into coordinates for the hand pre-shaping;
- object reaching;
- touch recognition with the object and slippage detection;
- evaluation of the forces to be applied during grasping and reactions to slippage events.

This organization can be replicated on a prosthetic device by organizing the control strategy in levels. In particular, a high-level could decode movement information from the biological signals of the amputee, corresponding to PPC, V6A, MIP and PMd areas in the human brain (Section 5). At a middle-level, thanks to the information from the high-level, the prosthetic hand fingers necessary for the grip are moved, on the basis of the user intention, to start the reaching and preshaping phases. Similarly, in the human brain, the IPLs, F5 and PMv areas are activated during preshaping, while the core region in the dorsomedial pathway and some regions between dorsomedial and dorsolateral code are responsible for the reaching phase (Section 5). The use of force sensors on the prosthetic hand allows measuring the grip force and detecting object slippage. They have the same role as SA-I, SA-II, and FA-II (Section 5.1) in the human hand. Once in contact with the object, the human hand modulates the grip force and continuously checks that the object does not fall, reacting if slippage events are detected (Section 5.2). To replicate this behavior, a low-level control lets the prosthetic hand to detect the first contact with the object, and to automatically adjust the grasping force, increasing it during the object slippage.

## 6. Control Strategies for Hand Prostheses

Over the years, several studies were performed to return good hand functioning to amputees. The first attempts at control strategies for hand prostheses date back to the 1960s when the different prototypes were developed with electronic hardware or logical-programming solutions [103]. Although knowledge of the brain was scarce at that time, it nevertheless proved sufficient to develop multiple-layers or hierarchical control strategies inspired by the distinct phases of the prehension [104,105]. The aim of this section is to analyze the selected papers to evaluate the multiple layers/hierarchical structure of control strategies, and the inspiration of each part in the functioning of the human hand.

The Southampton Adaptive Manipulation Scheme (SAMS) is the evolution of a work born in ’60 and expanded in the following by other researchers by adding new functionalities. In the ’60, at the University of Southampton, a group of PhD students researched the control of prosthetic hands. Their intention was obtaining a control more similar to a human hand despite a device with a limited number of DoFs independently controlled. In 1973, Codd, Nightingale, and Todd [106] proposed their solutions by introducing a hierarchy of control systems made of three levels. The lower level (reflex system) is automatic and independent by conscious intervention and generates a fast reflexive action. The intermediate level (intermediate system) intervenes in object shape decision, grip configuration and force control, receiving sensory information from the motor, accordingly with the sensory mechanism explained in Section 5.1 and the grasp stability of Section 5.2. The last level (supervisor system) receives command signals from the user, interpreting them in signals for the lower levels (in the human brain PPC, V6A, MIP and PMd are devoted to this task, Section 5). This strategy presents a hierarchical structure whereby each part is related to a specific task, as it happens in the human brain (Section 5), but lacks a reach phase. In 1985, Nightingale [107] expanded the concept of hierarchical control by introducing a microprocessor to work as a coordinator between the user and the prosthesis. Feedback about the position and the force for each drive is given by sensors such as encoders and strain gauges, as well as the peripheral neural loops that receive information from the muscles during a contraction to obtain a fine control during a movement (or a grasp). To switch from one activity to another, the human hand involves various groups of muscles, commanded by neural signals from the central nervous system (CNS). To achieve a similar behavior, the intermediate level was split into two subsystems: the ‘posture logic’ and the ‘force logic’. The first subsystem selects the motor drive for the movement chosen by the user. The use of the hand is simple for the user: for example, the user sends the command to close the hand and the hand automatically adapts its shape around the object. The second subsystem, called ‘force logic’, regulates the force when the object is grasped. In addition, the user can select a function among ‘touch’, ‘hold’, ‘squeeze’, ‘maneuver’, and ‘release’ and the force controller automatically adjusts the input to the drive involved during the grip phase. The force levels and the adjustments are automatically controlled to reduce the burden for the user (the same behavior in the human brain as explained in Section 5.1 and Section 5.2). A ‘command logic’ level was introduced to discard the use of the EMG signal for the proportional control to use it as a multilevel discrete switch; in this case, after the muscle contraction, the level interprets the EMG signal as input of the below level. In 1991, Chappell and Kyberd [108] described the transition among the functions (here called states) flat hand, position, touch, hold, squeeze, release, and link. The EMG signal from two antagonistic muscles (i.e., extensor and flexor carpi radii) are used to form a bipolar signal sent to the microcontroller (that also receives information about position, touch, forces, and slip). After the power or reset input has been sent, the first state is a flat hand. Starting from this state, the user performs the flexion to enter in the chosen position, the extension to return in the previous state. After contact with the object surface, the controller moves to the touch state. With a flexion, and after overcoming a threshold value, the controller goes into the hold state (in which the force control is activated). If the applied force is not sufficient and the object slips, an automatic force increment occurs. Another flexion signal places the controller in the squeeze state. Conversely, to release the grasped object an extension signal is necessary for returning in the position state. The user can choose from a set of hand postures (three types of precision, fist, small fist, side, flat hand), based on Napier’s classification [109]. With a sequence of signals (full flexion, full extension, full flexion, and relaxation) the hand adopts the full tips posture (where the thumb is abducted and opposes the tip of the index digit) and the user, with the sequence flexion-relax signal, switches the controller in the link state (a transaction state) where can select the precision states P1 or P2 (P1 where middle, ring and little fingers are flexed and the others are available for the grasp while P2 foresees middle, ring and little fingers extended and allows the same grasp as P1). The controller intervenes, thanks to sensor information, if the hand posture is unsuitable for a specific task (for example, if the user chooses precision and the controller receives information about the touch from the sensors on the palm, the controller moves in the first posture). In 1994, Kyberd et al. [110] added a new state (called PARK) to power the hand off when unused.

A validation of this strategy was carried out with a subject with congenital, below-elbow right hand loss who usually used a split hook. He was equipped with a laboratory version of the original Southampton Hand [111] with the SAMS control and a conventional proportional myoelectric to perform a comparison among the three prostheses. After a training phase to familiarize him with the myoelectric and the SAMS controls, the subject performed positional tasks, consisting of moving abstract objects from lower shelves to upper ones and vice versa, and practical tests consisted of abstract tasks and simulated real tasks (based on those devised by the Department of Health and Social Security (DHSS), Table 1), in the United Kingdom to assess artificial limbs [110]). Task times were recorded and an independent, experienced observer assigned scores comparing the hands, each with their control, with the hook (1. The hand was inferior to the split hook, 2. The hand was as successful as the split hook, 3. The hand was superior to the split hook).

The Southampton Hand with the SAMS control, the hook, and the two-channel Viennatone MM3 with a conventional myoelectric hand worked equally well for the larger abstract prehension tests. However, the standard myoelectric hand showed grasp limitations for small objects. The SAMS control with the Southampton Hand was able to adapt to the real object shape during grasping the contrary to the proportional control on the conventional myoelectric hand. The hook exhibited limitations with the largest object due to a small grasp capacity. Moreover, for the user it was very tiring to sufficiently open the hook to grasp large objects. The SAMS control with the Southampton Hand did not show these drawbacks and has been superior in performance (rating of an independent, experienced observer, Table 1) than to the hook in over half of the tasks (especially in power grip with large grasp) and equal in the rest, despite the execution time. The proportional control on the conventional myoelectric hand behaved similarly to or worse than the hook. Results are reported in Table 1.

In 1987, Tomovic, Bekey, and Karplus [112] developed a control strategy based on the reflex arc [113]. This strategy can be described in four phases:

1. Creation of a small number of geometric primitives to represent target objects with arbitrary shapes.
2. Pre-shaping and alignment of the hand to select the appropriate primitive (AIP, IPL, F5, and PMv in the human brain, Section 5).
3. Reduction of hand configurations to a limited number of standard configurations for grasping tasks.
4. Separation of the grasping in target approach phase and shape adaptation phase, with reflex control application (Section 5).

The reflex control principle is based on the activation of the movement patterns by specific sensory input and the subsequent completion of the movement without other intervention from nervous system higher centers (Section 5). This principle assumes that most reaching and grasping tasks in humans are derived from experience. During the target approach phase, the hand performs a reorientation and a pre-shaping to make easy the grasp. This phase is divided into target identification—where the objects identified by means of a vision system are replaced by geometric primitives, such as cylinders, cones, parallelepipeds, spheres—hand structure, and grasp mode selection to choose the involved fingers in the grasp. When the hand touches the object, the target approach phase ends, and the grasp phase starts. In this phase, an automatic shape adaptation is possible, employing control allowing a force selection related to the coefficients of friction between finger material and the object surface, and slippage sensing, with an increase of the forces until slippage stops. Force and slippage information are derived from sensors positioned on the fingers. The hand is provided as input for the task selection, but the authors did not specify the procedure to obtain it, while the knowledge base (containing shape, orientation, grasping, etc.) for the target approach phase is obtained from studies on human subjects performing several approach and grasping tasks, with a variety of positions and orientations. This structure is similar to the human brain levels described in Section 5. Moreover, the force and slippage management takes up the tactile sensory mechanism (Section 5.1) with the use of touch and slippage information to stably grasp an object (Section 5.2).

The Belgrade hand [114] has five fingers but only two motors, allowing a three-finger mode and a five-finger mode. The hand was equipped with touch and slippage sensors and then mounted on the PUMA 560 manipulator. There are no protocols and results because the paper is only focused on the control strategy.

In 2006, Cipriani presented a control strategy composed of two parts: the first one was devoted to high-level control and the second one focused on low-level control [115]. The high-level decodes the intention signals of the user used to choose the desired grasp and forces. The selection of grasp is possible among cylindrical, spherical, tri-digital, and lateral [116] and force between power or light. The low-level is composed of two subparts: pre-shaping and grasping phases. During pre-shaping all fingers are involved while in the grasping phase only the fingers chosen by a table (correlating grasp types and involved fingers) and grasp forces, are involved. After the pre-shaping phase, the desired force is selected. In the grasping phase, the hand closes the fingers using force control algorithms until the reaching of the global force. A global force error (about the total grip) and the finger force error are evaluated. The global force is calculated as the sum of the desired finger forces involved in the grip. Each finger can grip the object with the same force (the global force divided among the fingers involved in the grasp) but if a finger closes without touching the object, the global force is redistributed among the rest of the involved fingers, with a safety margin that stops the finger to avoid a finger break. In this strategy, low-level corresponds to the AIP, IPL, F5 and PMv areas (Section 5).

An underactuated five-finger with 16 DoFs (three for each finger plus one for the thumb opposition) with only six active DoFs (F/E for each finger and the thumb opposition) has been used. The force information is derived from strain gauges sensors placed on the tendons. Five able-bodied subjects have been equipped with the hand assembled on an orthopedic splint to reach and grasp different objects and the grasp (Table 2) has been considered successful if the object was stably held.

Experiments showed that the control is stable and after a disturb resulting in force distribution, and the hand returns to a stable grasp in a short time. The control strategy allowed performing stable grasps in 96% of the whole experiments (Table 3).

In 2012, Pastulosa presented a control strategy consisting of four parts: pre-shaping, closing, force control, and detection stages [117]. In the pre-shaping stage, the user can select the desired hand configuration among four possibilities: cylindrical, tip, lateral, and open hand (corresponding to AIP, IPL, F5 and PMv areas, Section 5). After this phase, the hand closes with the maximum velocity until contact with the object. This is the closing stage in which the velocity derivative is computed to determine the touch and then the activation of the stage. After contact with the object, the force control is activated, and the modulation of the force exerted on the object surface is possible (Section 5.2). This stage is alternated with the detection stage, activated when the stable grasp is reached Section 5.2). In this stage, to detect the possible object slippage, the information from the derivatives of the force sensor resistor (FSR, for detecting disturbances) and resistive flex sensors (RFS, for the detection of the unintended object) signals are used (as well as FA-I and SA-II are used in the human hand, Section 5.1). If the slippage is detected, in the force control stage the reference force is increased (with an empirical increase). The force reference of 1 N is empirically determined as a trade-off between object deformation and initial slippage. With a force within 5% of the reference value, the motor is turned off to reduce power consumption, a possible oscillatory behavior and to prevent overshoots because the response of each finger is slowed down.

The five-finger prototype has ten DoFs (only three actives for the F/E of thumb, index and the rest of fingers) allowing five grasping configurations: five-finger pinch, transverse volar grip, spherical volar grip, pulp pinch, and lateral pinch [118]. FSR (Interlink Electronics) sensors were placed on the tips and RFS (Spectra Symbol) were placed at the dorsal part of the thumb, index, and middle fingers. Two experiments were carried out to test the response to perturbations. In the first one, an aluminum cylinder was attached to a mass hanger system through a dual-range force sensor. The hand grasped with transverse volar grip and pultp pinch an object for about 20 s and different weights were placed on the base of the hanger. The experiment was repeated seven times for each weight. A motion sensor (Vernier MD-BTD) was used to measure the displacement of the object. To verify the ability of this strategy to modulate the applied force when a rotational force is applied, the hand grasped with a five-finger pinch a plastic lid attached to a fixed axle connected to the hanger system and the force sensor. Different weights were placed on the hanger base to produce different torques.

In all the experiments, the hand was able to quickly adjust the force during grasp to avoid object dropping. The maximum average displacement of the transverse volar grip experiments was 7.6 mm ± 2 mm while the same one of light weight objects was within the resolution of the motion sensor, i.e., 2 mm. The displacement for the pulp pinch configuration was 3.05 mm. In addition to small weights, the average displacement was less than the precision of the sensor. During the torque experiments, the control strategy was able to modulate the grasping force with no significant angular displacement. Indeed, the maximum average angle displacement was 10.7 degrees when 11 N ·cm of torque was applied.

In 2017, Quinayàs proposed a hierarchical human-inspired architecture [119]. The architecture levels are described below. The Human–Machine Interface (HMI) is devoted to measuring and interpreting the humans’ signals for identifying four types of grip postures (rest, open hand, power grip, and tripod grip) [120] and to sending this information to the next level (as well as the AIP, IPL, F5 and PMv areas in human brain, Section 5). The Haptic Perception (HP) level receives information from robotic hand sensors and HMI and generates information (contact and slip) to the high-level control (HLC), (FA-I and SA-II, Section 5.1). A contact is identified by imposing a minimum threshold to differentiate between noise and actual contact with the object. With a first-order time derivative of the force, slip can be detected. HLC receives information from HP and HMI and coordinates the execution order of the motor programs for the user task, by sending the commands to the mid-level control (MLC). Moreover, HLC also shares information with the learning module involved to acquire new behaviors and store recently learned information. MLC receives information from the above levels and generates a low-level command (LLC) and shares information, as joint positions and motor primitives, with the knowledge database and sends newly learned facts to be stored in a memory. The motor programs are: Repose, the default state of the hand; Pre-shaping, the hand is configured on a primitive to prepare object grasping; Grasping, a PI force control strategy (when the HLC detects the contact) is executed to obtain a stable grip without slippages; Slip, 10% of the force proportionally increases to contrast the slippage event (when the HLC detects the contact); Release, the hand completely opening; Point finger, the hand with the extended index finger; Reaching, the forearm movement to reach the object; Wait, the standby state in which an action is executed. The LLC level receives information from the hand sensors and generates the commands for the hand actuator and the patient sensory feedback system. A PID position control is used for tracking the trajectories in the pre-shaping phase and a PI control to maintain the desired force in the grasping phase. Furthermore, in this structure the sub-division is inspired to the brain areas (Section 5) but the reaching is not referred to the fingers onto the object. The prototype prosthesis hand UC2 is composed of three fingers (each finger has three phalanges) and nine DoFs: flexion/extension and also the opposing/repositioning for the thumb [121]. The hand is equipped with FSR sensors on the fingertips, covered with silicone. Two different validation tests were carried out: in the first one, a no-amputee subject has performed the object grasp to real-time monitoring of the performance of the different modules of the architecture; in the second one, a no-amputee subject has performed grasping of a cylindrical object of 190 g. A supplementary weight was added to simulate the slippage. This work only presented a control strategy without testing it on amputees.

All of the aforementioned strategies present common levels like the brain areas, as described in Section 5 and use information about force, touch, and slippage (Section 5.1) to stably control the object during grasping.

## 7. Discussion

The control strategies summarized above (Table 4) show a subdivision in states inspired by the human hand: the choice of the hand initial configuration is managed by a high-level, pre-shaping is obtained using predetermined forces and position values [122], corresponding to the AIP, IPL, F5, and PMv areas (Section 5), touch (FA-I, Section 5.1) and control of force and slippage (SA-II, Section 5.1) are completely automatic (Section 5.2), lightening the cognitive burden of the amputees during the grasping task.

The studies [106,107,108,110,112,115,117,119] presented similar solutions in terms of phases of prehension and management of forces and slippage events.

The SAMS strategy [106,107,108,110] replicates the behavior of motor control in the CNS. Amputee subjects can choose the grasp by muscle contractions and go through the various states. Once the configuration was chosen, with a muscle contraction, the hand starts the movement until the touch with the object activates the control to regulate the interaction force and manage the slippage, by incrementing the force during the event. In this phase, the user can increment the force or release the object. This strategy has a limited number of states allowing the user not to have a high cognitive burden and to avoid managing force and slippage without feedback. In contrast, the transition between states is possible through muscle contraction and co-contraction, an unnatural behavior.

The reflex control strategy [112] is based on the reflex arc: sensorial inputs to the brain allows complex cognitive and computational processes such as trajectory planning, pattern recognition, hand structure, etc., and produce signals to muscles to obtain the desired movement. The various shapes of the objects are reduced to a small number of geometric primitives. The hand, after the configuration has been chosen, can align and orient itself to the object. After the touch, the hand adapts itself to the object shape by controlling the interaction force and incrementing it during the slippage. The simplification of the configuration possibilities and an adaptable control seem to result in a light commitment for amputee patients.

The two-phase bio-inspired control strategy [115] allows a choice of configurations and forces directly from a table. After the configuration has been chosen, the hand will close touching the object, until the force references (both total and for each finger) are reached. The strategy allows opening and configuration choice, leaving the rest to the automatic control that independently closes the fingers and regulates the forces. In the pre-shaping phase, the choice of the fingers involved in the grasp is possible. The table is limited to only two force levels for the four possible configurations.

The neural network-based control strategy [117] allows the selection of the hand configuration among five possibilities covering the most commonly performed grasps. After the configuration choice, the hand closes with the maximum velocity until the contact with the object. The touch activates the control automatically regulating the grasp force. The slippage management occurs just when a stable grasp is reached. A possible slippage before the stable grasp is not detected by the control.

The hierarchical human-inspired architecture [119] is based on both the task planning paradigm and the imitation of the CNS behavior. The subject can choose from four configurations, then the hand will adjust according to the primitives. Successively, the hand closes and after the touch of the object, the control regulates the force and manages the slippage. Reaching is the forearm movement to achieve the object.

The examined approaches show some common critical points:

- The subject, through EMG signals, can only choose the grasp type but the simplicity of this functionality requires special attention when the hand starts closing because the closure velocity applied before touch [124] could cause the object to go out of the grasping area [125];
- A reach phase where the subject can voluntarily control the fingers during the object approach is missing (Section 5);
- Without a reaching phase, predetermined configurations are necessary for the pre-shaping phase not usable with a great number of objects (or shapes) [126];
- Except for the SAMS, in the other approaches the increase of the force during the automatic control is not possible;
- The force reference is obtained based on tests performed for one or a few levels of objects weights and they cannot be changed.
- A coordination strategy among the fingers to ensure the grasp stability is missing.

**Figure 3:**
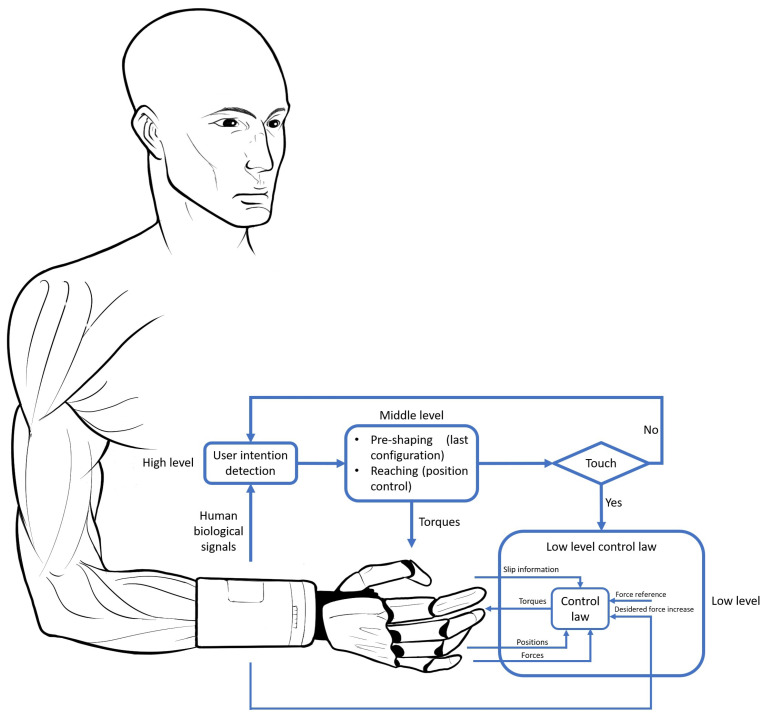
New solution inspired by human hand behavior.

To overcome these critical issues, a new control strategy based on the human hand should have distinct phases inspired by the neurophysiological subdivision of the brain, with a continuous presence of the user who can intervene at any time. A possible solution is presented in Figure 3.

At the high-level (corresponding to AIP, IPL, FR and PMv areas, Section 5.1), the human biological signals (EMG are typically used [127], the feasibility of the classification of electroneural signals was demonstrated [128]) are acquired and processed, with different techniques, to extract information about the desired movement. In this phase, the correlation between the decoded movement and the natural way the user performs is very important, to ensure the intuitiveness of the gesture. The choice of the movement is obtained by decoding the human signals and can be changed every time and in each phase, to replicate the user intention of switching the configuration during the grasp.

In the middle-level (corresponding to AIP, IPL, FR, PMv areas, the core region in the dorsomedial pathway and some regions between the two pathways code reaching information, Section 5), the pre-shaping activates only the involved fingers and then close them with a gradual increment of the position, up to reach the object surface (position control performed by the user) [126]. Analogously, in this phase it is also possible to open the hand.

Tactile information is important during object manipulation, as described in Section 5.1 By miming FA-I afferents (Section 5.1) when the touch between the object and the involved fingers (obtained from force sensors information) is detected, automatic control at a low level is activated. The control regulates the forces during the grasp and avoids the object slippage [129] using additional information from sensors and/or algorithms (Section 5.2). Moreover, as SA-II afferents (Section 5.1), slippage detection for a prosthesis is important to prevent the object fall since, in the absence of feedback, the amputee cannot modulate strength to counteract slippage [32]. The use of the only normal component of the force allows detecting the slippage in a few milliseconds [130].

The weight perception is provided by both the proprioception feedback and cutaneous cues [131]. The restriction of one of them tends to reduce the perception of the object weight [132]. The overall force reference can be obtained by combining forces and torques information obtained from a 6-axis sensor mounted on the wrist and from the sensors positioned on the fingertips. From the touch and in a few cycles of the automatic control, the mass could be determined of the object and to update the overall force reference to adapt it to the real value of the grasped object. Then, the overall value of the force reference can be redistributed to each finger according to its contribution during the grasp, as in the human hand [133].

To guarantee grasp stability (Section 5.2) and to use the opposition DoF of the thumb not only in the pre-shaping phase, an approach for controlling the fingers in a coordinated manner based on the virtual finger concept [133] can also be used. The approach takes into account the normal forces acquired by the sensors on the fingertips to calculate the torque to reposition the thumb during a slippage event.

Although these actions are automatic, the user can intervene if they want to increase the force. For instance, by using a hierarchical classification approach to assess the desired hand/wrist gestures, as well as the desired force levels to exert during grasping tasks [134], the user adds an input value to the reference force; the maximum force increment, built on the correspondent maximum human signal performed, is limited in a safe range to avoid the object and prosthetic breaking. The human signal relaxation leads to the subtraction of the previous increment until return to the initial reference value when the signal activity is close to zero. If the user wants to open the hand with the automatic control, they perform the corresponding signals, and the prosthetic hand opens the involved fingers. After losing the touch signal, the control returns to the medium level; the opening occurs as the closing, but the user could re-close the hand starting a new grasp. However, the return to the open hand configuration to choose a new configuration or a new grasp is not necessary.

## 8. Conclusions

The human hand is a complex system studied for thousands of years, and still fascinates many researchers in different fields. Replicating its correct functioning in a prosthesis [135] is still an open challenge. Actual commercial myoelectric prosthetic hands are simple devices allowing the opening and the closing, as a gripper [136], by using two antagonistic muscles [137], such as flexion and extension of the wrist [138]. An unnatural and not intuitive behavior and for this reason, many amputees use cosmetic prostheses [139,140].

To replicate a human hand with a device is necessary not only as regards the external aspect but also the functions [141]. In recent years, there were several attempts but all without a complete replication of the human hand [142]. Thanks to neuroscience, study related to the management of the functioning of the human hand by the nervous system has been possible and sufficient to start to develop a control strategy replicating its functions [143].

The strategy presented in this work is based on the subdivision of the prehension in the brain, pre-shaping, reach and grasp, and on the managing of the tactile information to ensure grasp stability. Each part is not completely independent because an overlapping among the areas in the brain is present [144,145]. Moreover, the user can intervene at any time by assuming complete control, to stop or change the movement, as in human behavior, without waiting for the end of the command in progress.

In recent years, new techniques were developed allowing the creation of new muscle units to obtain more precise biological signals for the high-level [146,147,148]. This aspect linked to surgery would allow increasing the quality of control overcoming the limitations of current technologies [149] promoting the use of parallel-decoding-strategies of intentional-movements information from EMGs [150].

A prosthetic hand is a device with many actuators and sensors [151] but in the absence of a control strategy able to replicate the human behavior of the hand, it will be a tool that can be never accepted by those who have lost a so fundamental and versatile part of the body [22,137].

## Figures and Tables

**Table 1:** Comparison of three controls (RH: Right hand—LH: Left hand) [110].

	Time (s)			Rating	
**Task**	**SAMS**	**Hook**	**Myo**	**SAMS**	**Myo**
**Cutting**					
Fork LH Knife RH	57	26	-	2	1
Fork RH Knife LH	49	42	-	2	1
**Change grip, Spear to scoop**	12	14	-	2	1
**Open bottle and pour**					
Top LH Bottle RH	26	29	-	3	1
Top RH Bottle LH	11	12	12	3	1
**Carry tray**	21	17	18	2	2
**Cut slice of bread**					
Loaf LH Knife RH	41	42	-	3	1
Loaf RH Knife LH	17	26	17	3	2
**Butter bread**					
Bread RH Knife LH	16	19	20	2	1
Bread LH Knife RH	36	31	29	3	2
**Fasten belt**	32	29	31	3	2
**Toothpaste onto brush**					
Brush LH Tube RH	36	21	20	3	2
Brush RH Tube LH	42	-	15	3	2
**Grasp telephone receiver**	19	5	5	3	2
**Grasp pen and write**	30	20	22	2	2
**Cigarette from pack**					
Pack LH Cig RH	28	20	44	2	2
Pack RH Cig LH	12	11	13	2	2
**Use mallet and chisel**					
Mallt LH Chis RH	16	11	9	3	3
Mallt RH Chis LH	18	-	15	3	3
**Task**	**SAMS**	**Hook**	**Myo**	**SAMS**	**Myo**
Pick up coins	34	17	27	3	2
**Lift and pour kettle**	29	15	-	3	1
**Tear and fold paper**	46	46	26	2	2
**Put paper in envelope**					
Paper LH Env RH	19	18	22	2	2
Paper RH Env LH	13	18	20	2	2
**Grasp cup**	8	6	7	2	2

**Table 2:** Grasp type and objects involved during the tests [115].

Grasp Type	Object	Size (mm)	Weight (g)
**Cylindrical**	Small Bottle	Ø = 60	750
	Big Bottle	Ø = 85	1500
	Cylinder	Ø = 70	100
	Cylinder	Ø = 50	500
	Cylinder	Ø = 50	50
**Spherical**	Rounde sponge	Ø = 100	30
	Sphere	Ø = 60	120
**Tri-digital**	Sphere	Ø = 35	20
	Sphere	Ø = 45	25
	Sphere	Ø = 55	30
	Felt-tip pen	Ø = 20	70
	Mobile phone	Ø = 40	200
	Cube	Ø = 50	80
**Lateral**	Postcard	1	10
	Key	2	80
	Floppy disk	3	40
	CD	1	30

**Table 3:** Results about different tasks [115].

Grasp Type	Cylindrical	Spherical	Tri-Digital	Lateral
N° objects	5	2	6	4
N° trials	5	5	5	5
Successful rate	25/25	10/10	27/30	20/20
Global successful rate = 82/85				

**Table 4:** Summary of the reported analysis.

Study	Control Strategy Subdivision	Robotic Hand	Force Sensor	Slippage Detection	Touch Detection
SAMS [106,107,108,110]	1. Automatic loop 2. Intermediate 2.1. Posture logic 2.2. Force logic 3. Command logic	Laboratory version of the original Southampton Hand [111]	Force-sensitive, resistive sheet	Microphone [108,123]	Light-emitting diode, phototransistor [108,123]
Reflex control strategy [112]	1. Target approach phase 1.1. Target identification 1.2. Hand structure 1.3. Grasp mode 2. Grasp phase	Belgrade hand [114]	Not specified	Not specified	Not specified
Two-phases bio-inspired control strategy [115]	1. High level 1.1. Decoding of user intentions signals 1.2. Choosing desired grasp and forces 2. Grasping task 2.1. Pre-shaping 2.2. Grasping phase	Underactuated five-finger with 16 DoFs (three for each finger plus one for the thumb opposition) with only six actives (F/E for each finger and the thumb opposition)	Strain gauges sensors on tendons	Not specified	Not specified
Neural Network-Based control strategy [117]	1. Pre-shaping 2. Closing 3. Force control 4. Detection stage	Five-finger prototype hand has 10 DoFs (only three actives for the F/E of thumb, index and the rest of fingers)	FSR	The derivative of FSR e RFS	Derivative of RFS
Hierarchical human-inspired architecture [119]	1. Human–Machine Interface 2. Haptic Perception 3. High-level control 4. Mid-level control 5. Low-level command	UC2 [121] is composed of three fingers (each finger has 3 phalanges) and 9 DoFs: flexion/extension and the opposing/repositioning for the thumb	FSR	The negative derivative of the force signal	The positive derivative of the force signal
